# Imatinib and Nilotinib increase glioblastoma cell invasion via Abl-independent stimulation of p130Cas and FAK signalling

**DOI:** 10.1038/srep27378

**Published:** 2016-06-13

**Authors:** Antonina Frolov, Ian M. Evans, Ningning Li, Kastytis Sidlauskas, Ketevan Paliashvili, Nicola Lockwood, Angela Barrett, Sebastian Brandner, Ian C. Zachary, Paul Frankel

**Affiliations:** 1 Centre for Cardiovascular Biology and Medicine, Division of Medicine, The Rayne Building, London WC1E 6JJ, United Kingdom; 2 Division of Neuropathology, Institute of Neurology, London WC1E 6JJ, United Kingdom; 3 UCL COMPLEX, University College London, London WC1E 6JJ, United Kingdom

## Abstract

Imatinib was the first targeted tyrosine kinase inhibitor to be approved for clinical use, and remains first-line therapy for Philadelphia chromosome (Ph+)-positive chronic myelogenous leukaemia. We show that treatment of human glioblastoma multiforme (GBM) tumour cells with imatinib and the closely-related drug, nilotinib, strikingly increases tyrosine phosphorylation of p130Cas, focal adhesion kinase (FAK) and the downstream adaptor protein paxillin (PXN), resulting in enhanced cell migration and invasion. Imatinib and nilotinib-induced tyrosine phosphorylation was dependent on expression of p130Cas and FAK activity and was independent of known imatinib targets including Abl, platelet derived growth factor receptor beta (PDGFRβ) and the collagen receptor DDR1. Imatinib and nilotinib treatment increased two dimensional cell migration and three dimensional radial spheroid invasion in collagen. In addition, silencing of p130Cas and inhibition of FAK activity both strongly reduced imatinib and nilotinib stimulated invasion. Importantly, imatinib and nilotinib increased tyrosine phosphorylation of p130Cas, FAK, PXN and radial spheroid invasion in stem cell lines isolated from human glioma biopsies. These findings identify a novel mechanism of action in GBM cells for two well established front line therapies for cancer resulting in enhanced tumour cell motility.

Abnormal or dysregulated tyrosine kinase (TK) activity represents a large proportion of oncogenic activity across a broad range of cancers. TK mutation, enhanced expression and autocrine stimulation can lead to downstream signalling that is responsible for enhanced migration, proliferation, angiogenesis and survival of cancer cells^1^,^2^.

Given their cardinal role in tumourigenesis, TKs have been the target for the development of inhibitors as therapeutics. The constitutively active oncoprotein BCR-ABL tyrosine kinase is the driver of Philadelphia chromosome (Ph+)-positive chronic myeloid leukaemia (CML)^3^. Imatinib, a BCR-ABL inhibitor (Gleevec, Novartis Pharmaceuticals Corporation, East Hanover, NJ), was the first selective tyrosine-kinase inhibitor (TKI) to be approved for the treatment of a cancer in 2002^4^. Imatinib is currently first-line therapy for Ph+-CML leading to remission in the majority of CML patients, and is also used for treatment of other malignancies including gastrointestinal stromal tumours (GIST). Imatinib was developed to bind to the ATP-binding pocket of BCR-ABL, competing with ATP, and thus blocking kinase activity^1^,^2^. Nilotinib, a second generation TKI, shares a very similar target spectrum with imatinib and was approved in 2010 to provide second-line treatment in case of resistance or intolerance to imatinib^5^. However, a considerable amount of CML patients do not respond favourably to nilotinib after imatinib treatment^6^.

Despite encouraging clinical results for CML, and for GIST^7^, imatinib has failed clinical trials for glioblastoma, where it shows no significant inhibition of tumour growth or extension of survival^8^,^9^ Imatinib and nilotinib potently inhibit tyrosine kinases including ARG, c-KIT, PDGFR and DDR1. Moreover, imatinib and nilotinib are reported to cause activation of intracellular kinases including the PI3K, Akt and ERK pathways^3^,^7^,^10^. Inhibition of other TKs and co-activation of signalling pathways may account both for the development of imatinib resistance in Ph + CML and GIST, and imatinib’s lack of efficacy in glioblastoma. The functional consequences of imatinib and nilotinib treatment on enhanced signalling in tumour cells remain poorly understood. In particular, their effects on cell functions modulating tumour behaviour are essential for understanding critically important aspects of drug treatment including non-responsiveness, the development of resistance, and the occurrence of side-effects.

The acquisition of enhanced cell motility provides tumour cells with the capacity to invade their surrounding tissue and metastasise, and is considered one of the “hallmarks of cancer”^11^. In this study, we demonstrate that imatinib and nilotinib treatment of glioblastoma and patient-derived glioblastoma stem cells results in increased tyrosine phosphorylation of several signalling proteins centrally important for cell motility including p130Cas, focal adhesion kinase (FAK), and paxillin (PXN), and strikingly increases tumour cell and stem cell migration and invasion. Surprisingly, these effects are independent of the known imatinib and nilotinib targets, ABL, ABL2 (ARG), c-KIT, PDGFRβ and DDR1.

Our findings point to a novel and important effect of imatinib and nilotinib upon tumour cell motility. These data may provide insight as to why imatinib has failed clinical trials for glioma, and have implications for understanding mechanisms underlying the development of imatinib and nilotinib resistance in other human malignancies.

## Experimental

### Cell culture

U87, U251, and U118 glioma cells were cultured in Dulbecco’s modified Eagle’s medium (DMEM) containing 10% (vol/vol) foetal calf serum (FCS) supplemented with Pen/Strep (1:100; P4333-Sigma).

### Derivation of Human GBM stem cell lines

All patients gave informed consent before the surgical intervention. The storage of human tissue is governed by the Human tissue Act (UK; HTA License #’s 12054). The use of tissue and cells has been approved by the National Hospital Ethics Committee (LREC 08/0077) and all methods were carried out in accordance with the approved guidelines. All tumours were diagnosed as glioblastoma (WHO grade 4) by neuropathologists. The samples were taken directly from the operating theatre and placed in cold Dulbecco’s modified Eagle’s medium/Ham’s F12 (DMEM/F12). The samples were finely minced, erythrocytes lysed by ACK buffer (Invitrogen) and tissue dissociated using Trypsin/EDTA. The resulting suspension was centrifuged and pellets re-suspended in DMEM/F12 medium supplemented with B27, bFBF, EGF and penicillin-streptomycin. Fresh medium was added to the cell suspensions every 3–5 days. When neurospheres formed, the suspension was transferred to flasks coated with laminin (Sigma). Adherent monolayer cells were sub-cultured by treatment with Trypsin/EDTA and plating them onto laminin coated plates for western blotting experiments or directly used for spheroid formation as described below.

### Antibodies, reagents and small interfering RNAs (siRNAs)

Antibodies to Phospho-p130Cas (Y410), DDR1, ABL1, ERK, Phospho-ERK (T202/Y204), Phospho-Paxillin (Y118), and Phospho-PDGF-R β (Y751) were from Cell Signalling Technology Inc., (Danvers, MA, USA). Antibodies to PDGFR-β, focal adhesion kinase (FAK; A-17), β1 Integrin, β3 Integrin, c-RAF, B-RAF, Paxillin (H-114), and glyceraldehyde 3-phosphate dehydrogenase (GAPDH; V-18) were from Santa Cruz Inc., (Heidelberg, Germany). Secondary antibodies to mouse, goat and rabbit were also from Santa Cruz Inc. Antibody to Phospho-FAK (Y861) was purchased from Life Technologies (Carlsbad, USA). Antibody to p130Cas antibody was from BD Transduction Laboratories (Oxford, UK). The antibody to ABL2 (N1N3) was from GeneTex (Irvine, USA). The Fak/Pyk2 inhibitor PF573228 was purchased from Tocris Bioscience (Bristol, UK). PDGF-BB was purchased from Peprotech (London, UK). Dimethyl sulphoxide (DMSO) was purchased from Sigma. U0126, Imatinib and Nilotinib were purchased from Source Bioscience (UK). The pH2B-GFP plasmid, encoding histone H2B fused to the green fluorescent protein, was obtained from Professor Sibylle Mittnacht (UCL Cancer Institute).

The following small interfering (si) RNAs were purchased from Dharmacon (GE Healthcare, UK):

si ITGB1-1: 5′-GAACAGAUCUGAUGAAUGA‐3′

si ITGB1-2: 5′-CAAGAGAGCUGAAGACUAU‐3′

si ITGB3-1: 5′-CUCUCCUGAUGUAGCACUUAA-3′

si ITGB3-2: 5′-CACGUGUGGCCUGUUCUUCUA-3′

si p130Cas #2: 5′‐GGUCGACAGUGGUGUGUAU‐3′

The following siRNA was purchased from Life Technologies (Carlsbad, USA):

si p130Cas #1: 5′ ‐GAGUUUGAGAAGACCCGATT‐3′

The following siRNAs were purchased from Qiagen (Crawley, UK):

AllStars Negative Control

siPDGFR-β 5′-GGAACGTGCTCATCTGTGA-3′

siABL1 (#10) 5′-ACGCACGGACATCACCATGAA-3′

siABL2 (#8) 5′-AACCCTGTCCTTAATAACTTA-3′

siCRAF (#5) 5′-AAGACGTTCCTGAAGCTTGCC-3′

siBRAF (#1) 5′-AACATATAGAGGCCCTATTGG-3′

siDDR1 (#9) 5′-ACGGTGTGAATCACACATCCA-3′

### siRNA Transfection

U87 and U251 glioma cells at 60% confluence were transfected with Lipofectamine 2000 (Invitrogen) using 25 nM final concentration of siRNA as described^12^.

### Immunoblotting

For immunoblotting, cells were lysed in a solution containing 50 mM Tris-HCl (pH 7.5), 1% Triton X-100, 150 mM NaCl, 5 mM EDTA, complete protease inhibitor (Roche) and phosphatase inhibitors I & II (Sigma) and analysed by SDS-PAGE using 4 to 12% Bis-Tris gels (NuPAGE; Invitrogen), followed by electrotransfer onto Invitrolon polyvinylidene difluoride membranes (Invitrogen). Membranes were blocked with 5% (wt/vol) non-fat dry milk and 0.1% (vol/vol) Tween-20 in Tris-buffered saline for 1 hour at room temperature, before being probed with the primary antibody by overnight incubation at 4 °C, followed by incubation for 1 hour at room temperature with a horseradish peroxidase-linked secondary antibody (Santa-Cruz) and detection using ECL reagents (Bio-Rad, Hercules, USA), following the manufacturer’s protocol. Immunoblots were quantified by scanning of films with a calibration strip and analysis by densitometry using Image J (US National Institutes of Health; http://rsb.info.nih.gov/ij).

### Immunofluorescent staining and Confocal Imaging

For immunofluorescent staining, cells were fixed in 4% paraformaldehyde in PBS for 60 min followed by permeabilisation in 0.2% Triton X100 for 30 min. Antibody incubations were performed overnight at 4 °C in 1% BSA, 0.1% Tween20 in PBS. Confocal imaging was performed using a LEICA SPE2 upright microscope running LEICA-LAS software using sequential imaging capture.

### Transwell chemotactic migration assay

This assay was performed as described previously^12^. Briefly, Transwell cell culture inserts (Falcon; BD Biosciences, Oxford, UK), were inserted into a 24-well plate. Serum free media supplemented with or without imatinib or vehicle were placed in the bottom chamber, and U87 glioma cells in suspension (1.5 × 10^5^ cells/well in serum free DMEM) were added to the top chamber and incubated at 37 °C in for 6 h. Cells that had not migrated or had only adhered to the upper side of the membrane were removed before membranes were fixed and stained with a Reastain Quik-Diff kit (IBG Immucor Ltd, West Sussex, UK). Cells that had migrated to the lower side of the membrane were counted in four random fields per well at 20× magnification using an eyepiece indexed graticule.

### Cell Proliferation assay

Proliferation of U87MG cells (stably expressing H2B-GFP) was determined in 96-well plates (seeding density of 2000 cells per well) by assessing total cell fluorescence intensity per well in living cells using an IncuCyte Zoom (Essen Bioscience) for up to 96 hours.

### Three dimensional (3D) spheroid invasion assay

Spheroids were generated using the metho-cellulose technique as previously described^13^,^14^. siRNA transfection were carried out on cells as described above. Following 24 hours transfection, cells were trypsinised, and 5 × 10^4 ^cells/ml were suspended in a medium containing a 4:1 (v/v) mixture of 10% FCS in DMEM and methylcellulose. Spheroids were produced by pipetting 100 μl of the cell suspension into a well of a 96-well round bottomed non-tissue culture plate and incubating for 24 hours (37 °C, 5% CO_2_). Spheroids were collected and embedded in Collagen I plugs (2.1 mg/ml) prepared from fibrillar bovine collagen I (3.1 mg/ml; PureCol) by dilution in DMEM in accordance with the manufacturer’s protocol (Nutacon, The Netherlands). The collagen I solution was supplemented with either DMSO or 10 uM imatinib or 1 uM nilotinib. Spheroids were allowed to invade for 48 hours followed by fixation in 4% formaldehyde. Spheriod Invasion was determined by measuring the circular area of the spheroid core and the rim of Invasion using Image J. The rim of invasion was determined by the circular distance from the edge of the core to the edge of contiguous invading cells^13^,^15^.

### Scratch wound assay

Cells were seeded to confluence, scratched evenly at the centre, and treated with imatinib, nilotinib, or vehicle control. Rate of wound closure was measured using an Incucyte Zoom (Essen Bioscience, UK). Images were captured every hour for 30 hours.

### Immunoprecipitation

Cells were washed with PBS, lysed in NP40 (50 mM Tris–HCl at pH 8, 150 mM NaCl, 0.5% NP40) containing protease and phosphatase inhibitors and centrifuged for 15min at high speed (16000 *g* at 4 °Cmin). Immune complexes were collected when 1mg of cell lysate was immunoprecipitated with 2 μg of antibody or with control IgG. Lysis buffer was used to wash the beads three times before a final wash using 0.6M lithium chloride was performed. These samples or 20 μg lysate was then supplemented with sample buffer (Tris at pH 6.8, 20% glycerol, 5% SDS, β-mercaptoethanol and bromo-phenol blue), separated by SDS–PAGE, transferred to a nitrocellulose membrane and then immunoblotted.

### Statistical analysis

The data displayed on graphs are means, with error bars representing the standard error of the mean (SEM). Statistical analysis was performed by two-way analysis of variance (ANOVA), or T-test where appropriate. P < 0.05 was considered significant.

## Results

**Figure 1:**
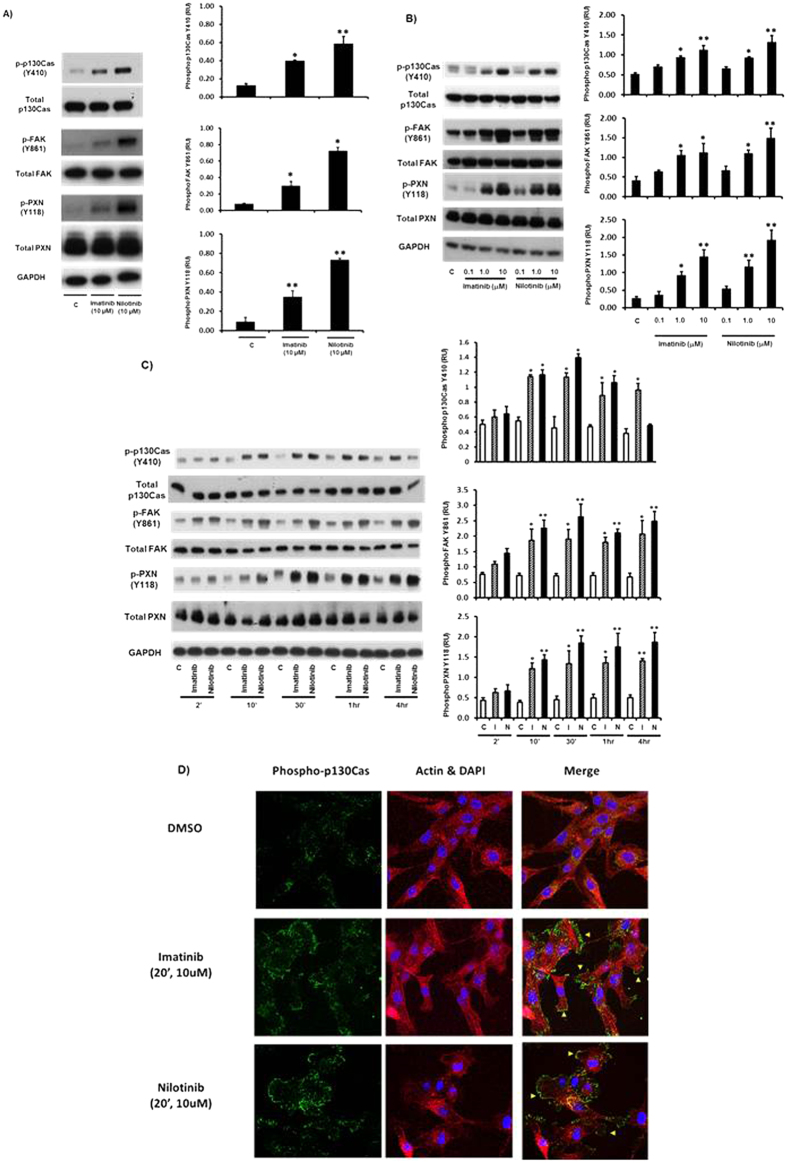
Imatinib and nilotinib treatment of human GBM cells leads to increased p130Cas, Focal Adhesion Kinase (FAK) and Paxillin (PXN) tyrosine phosphorylation. Cells (~80% confluent) were incubated in SFM for ~18 hr prior to treatment with either SFM & DMSO vehicle control (C), or imatinib or nilotinib at the indicated concentrations and times. Cell lysates were then prepared, blotted, and probed with the indicated antibodies. Blots shown here and in all subsequent figures unless indicated are representative of at least three separate experiments. (**A**), U87MG cells were treated with vehicle control (C), or 10 μM imatinib or 10 μM nilotinib for 20 minutes. (**B**), Dose dependency of imatinib or nilotinib treatment for 20 minutes in U87MG cells. (**C**), Time course of U87MG cells treated with vehicle control (C), or 10 μM imatinib or 10 μM nilotinib. Quantitation of tyrosine phosphorylation was performed by densitometry using Image J. In each panel, data from at least three independent experiments are presented as phosphorylation relative units (RU) (means +/− s.e.m.) normalized to total protein levels; *p < 0.05, **p < 0.01 compared to vehicle control (**C**). ^#^p > 0.05 compared to vehicle control (C). (**D**), U87MG cells were seeded on glass cover slips and incubated in SFM for ~18 h prior to for 20 min with vehicle control (C), or 10 μM imatinib or 10 μM nilotinib. Confocal imaging was performed as described in Materials and Methods, with phosphorylated p130Cas (Y410) staining in green, actin in Red and DAPI in purple. Images are representative of at least three separate experiments. Arrows point to areas of increased p130Cas (pY410) localisation to the membrane upon treatment.

We treated the human glioblastoma multiforme (GBM) cell line U87MG with either imatinib or nilotinib and investigated their effects on key signalling components required for tumour cell motility and invasion. Tyrosine phosphorylation of p130Cas at Tyr410, FAK at Tyr861 and PXN at Tyr118 play important roles in cell migration and invasion and were used as readouts of activation of these signalling pathways^12^,^16^,^17^. Treatment with 10 μM imatinib or nilotinib caused a striking increase in tyrosine phosphorylation of p130Cas, Focal Adhesion Kinase (FAK) and Paxillin (PXN) (Fig. 1A). These effects were dose-dependent, with a significant increase detected at 1 μM and a maximal response at 10 μM for both imatinib and nilotinib in U87MG cells (Fig. 1B). Because the peak plasma/serum concentrations of imatinib and nilotinib, are approximately 5 μM and 4 μM, respectively^18^,^19^, our data indicate that these effects occur at clinically relevant concentrations. The effects of imatinib and nilotinib on tyrosine phosphorylation of p130Cas, FAK and PXN were also rapid and sustained with a significant increase observed at 10 min, reaching a maximum at 30 min, which was maintained for up to 1 hour (Fig. 1C). After 4 hours, p130Cas tyrosine phosphorylation declined to near basal level, whereas FAK and PXN tyrosine phosphorylation remained strongly elevated. These results were further supported by the finding that imatinib and nilotinib induced p130Cas, FAK and PXN tyrosine phosphorylation in the glioma cell line, U251MG (Fig. S1).

We also examined the effect of imatinib and nilotinib treatment on the localisation of phosphorylated p130Cas in U87MG cells by performing immunofluorescence confocal microscopy. As expected the levels of phosphorylated p130Cas (Tyr 410) increased in both imatinib and nilotinib treated cells after 20 min. We also observed a striking redistribution of phoso-p130Cas to the cell membrane, localising along what appear to be membrane ruffles (Fig. 1D).

**Figure 2:**
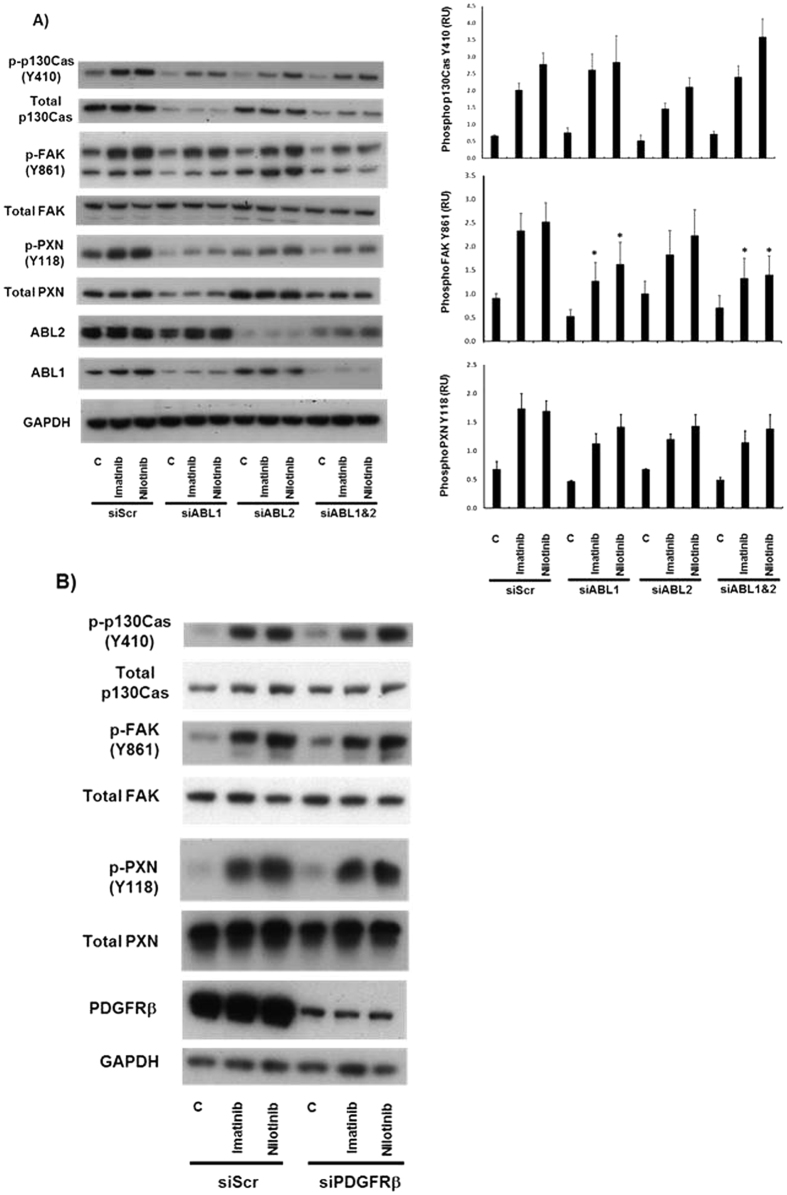
Imatinib and Nilotinib target proteins are not required for increased p130Cas, FAK and PXN tyrosine phosphorylation. (**A**), U87MG cells were transfected with siRNA targeting ABL1 (siABL1), ABL2 (siABL2) or together at a concentration of 25 nM, or with 25 nM of a control scrambled siRNA (siScr). (**B**) U87MG cells were transfected with siRNA targeting PDGFR Beta (siPDGFRB), cells were transfected at a concentration of 25 nM, or with 25 nM of a control scrambled siRNA (siScr). 48 hr post transfection, cells were incubated in serum-free medium (SFM) for ~18 hr prior to treatment with SFM & DMSO vehicle control (C), or 10 μM imatinib or 10 μM nilotinib for 20 minutes. Cell lysates were then prepared, blotted, and probed with the indicated antibodies.

Imatinib and nilotinib are both inhibitors of the Abl family tyrosine kinases, ABL1 and ABL2 (also called ARG). Furthermore, since Abl has been reported to regulate p130Cas tyrosine phosphorylation^20^, we reasoned that ABL1 and/or ABL2 could be mediating the effects of imatinib and nilotinib treatment in these glioma cell lines. We treated U87MG cells with siRNA to ABL1 and ABL2 either individually or in combination in the presence of imatinib, nilotinib or vehicle control. Knockdown of ABL1 or ABL2 alone or together had no effect on p130Cas, FAK and PXN tyrosine phosphorylation in control treated cells. Furthermore ABL1 and ABL2 silencing had no effect on imatinib and nilotinib mediated increases in p130Cas and PXN tyrosine phosphorylation, whereas tyrosine phosphorylation of FAK was reduced. Interestingly, knockdown of ABL1 and to a lesser extent ABL2 caused a decrease in total p130Cas and total PXN expression, whilst total FAK levels were unaffected (Fig. 2A). Imatinib and nilotinib inhibit additional tyrosine kinases, including platelet derived growth factor receptor beta (PDGFRβ), stem cell growth factor receptor (c-KIT), and discoidin domain receptor tyrosine kinase 1 (DDR1). However, silencing of PDGFRβ and DDR1 had no effect on increased tyrosine phosphorylation in response to imatinib and nilotinib (Fig. 2B & S2A). We were unable to detect c-KIT in U87MG cells, whereas we could readily detect c-Kit in human coronary artery smooth muscle cells (HCASMCs), indicating that c-Kit is not significantly expressed in U87MG cells (Fig. S2B).

The SRC tyrosine kinase is well known to play a role in the regulation of p130Cas, FAK and PXN tyrosine phosphorylation^20^. We therefore looked at the effect of using the SRC kinase inhibitor, PP2, in imatinib and nilotinib treated U87MG cells. We found that PP2 treatment leads to a complete abrogation of both basal and imatinib/nilotinib induced tyrosine phosphorylation of p130Cas, FAK and PXN (Fig. S3). This result led us to look at the levels of tyrosine 416 phosphorylation in SRC. We found that imatinib and nilotinib treatment did not result in any changes in the levels of Y416 phosphorylation (data not shown).

It has recently been reported that imatinib and nilotinib treatment leads to activation of MEK and ERK in several human tumour cell lines^3^, and ERK signalling has been implicated in meditating signalling pathways required for migration of glioma cells^21^,^22^. We therefore examined the effect of imatinib and nilotinib treatment on ERK activation in U87MG cells. Although imatinib treatment increased levels of phosphorylated ERK, nilotinib treatment had no effect (Fig. S4A). Furthermore, whereas the MEK inhibitor, U0126, completely abolished levels of phosphorylated ERK in all samples treated, it had no effect on imatinib and nilotinib stimulation of p130Cas, FAK, and PXN tyrosine phosphorylation (Fig. S4A). It was recently reported that imatinib and nilotinib can bind to B-RAF and C-RAF leading to the formation of RAF hetero- and homo-dimers, and stimulate paradoxical activation of BRAF and CRAF in the presence of activated RAS^3^. We therefore considered the possibility of a RAF-dependent, MEK/ERK-independent pathway leading to increased tyrosine phosphorylation of p130Cas, FAK and PXN. However, silencing of B-RAF or C-RAF either alone or together had no effect on imatinib and nilotinib stimulated increases in tyrosine phosphorylation (Fig. S4B). Because imatinib treatment was able to increase levels of ERK phosphorylation (Fig. S4A) we looked at the effect of imatinib and nilotinib treatment on U87MG cell proliferation. As shown in Fig. S7, drug treatment has no significant effect on cell proliferation.

Integrins are known to mediate p130Cas and FAK tyrosine phosphorylation through beta 1 & beta 3 subunits^20^,^23^. To test the possibility that imatinib and nilotinib stimulated tyrosine phosphorylation of p130Cas, FAK and PXN occurs via an integrin signalling pathway, we treated U87MG cells with siRNA to either beta 1 or beta 3 integrins. Knockdown resulted in a marked reduction in integrin protein expression, yet had no effect on increased p130Cas, FAK and PXN tyrosine phosphorylation (Fig. S4C,D).

**Figure 3:**
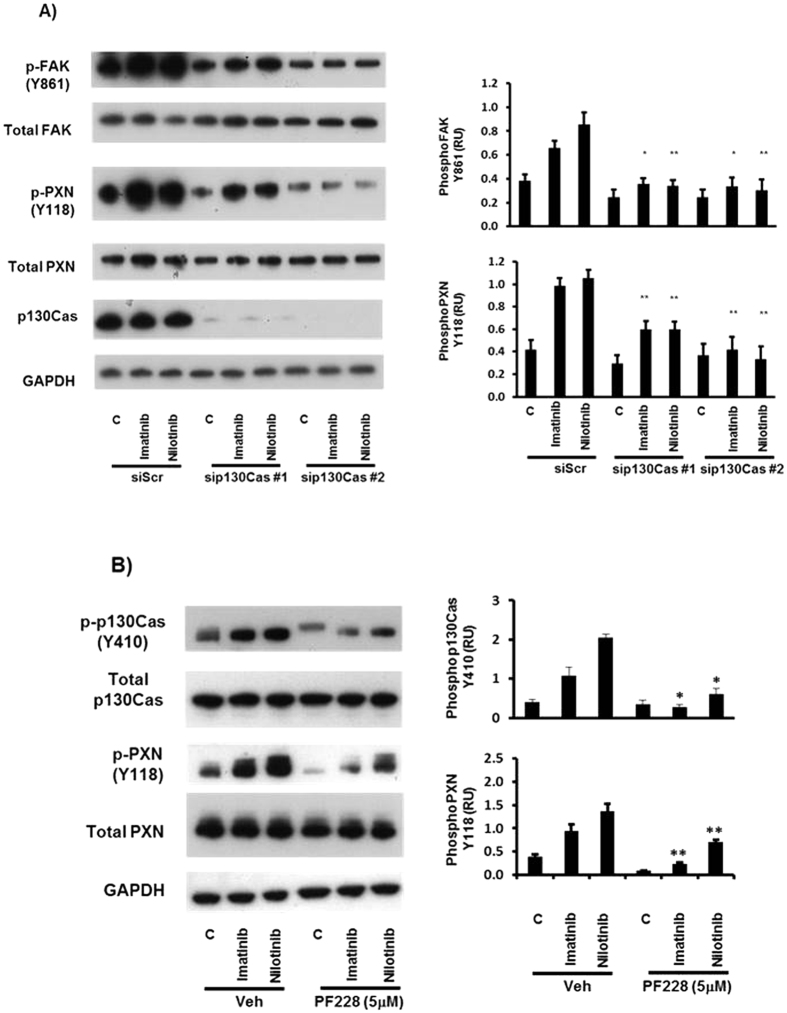
Increased p130Cas, FAK and Paxillin tyrosine phosphorylation is dependent on p130Cas expression and FAK kinase activity (**A**) U87MG cells were transfected with siRNA targeting p130Cas (si p130Cas) at a concentration of 25 nM, or with 25 nM of a control scrambled siRNA (siScr). 48 hr post transfection, cells were incubated in serum-free medium (SFM) for ~18 hr prior to treatment with SFM & DMSO vehicle control (C), or 10 μM imatinib or 10 μM nilotinib for 20 minutes. (**B**) U87MG cells (~80% confluent) were incubated in SFM for ~18 hr prior to pre-incubation for 30 min with 5 μM PF573228 or the vehicle (0.05% DMSO) (C) prior to treatment with SFM & DMSO vehicle control (C), or 10 μM imatinib or 10 μM nilotinib for 20 minutes. Cell lysates were then prepared, blotted, and probed with the indicated antibodies. Quantitation of tyrosine phosphorylation was performed by densitometry using Image J. In each panel, data from at least three independent experiments are presented as phosphorylation relative units (RU) (means +/− s.e.m.) normalized to total protein levels; *p < 0.05 compared to vehicle control (C).

Because p130Cas and FAK have been reported to exist in multi-protein complexes required for cell motility^20^, we examined the association of these molecules in glioma cells, and their interdependence in the response of U87MG cells to imatinib and nilotinib. Immunoprecipitation of p130Cas and subsequent immunoblotting showed that FAK and PXN were both constitutively complexed with p130Cas in U87MG glioma cells, and that these complexes were not affected by imatinib or nilotinib treatment (Fig. S5). However, targeted knockdown of p130Cas with two different siRNA significantly reduced tyrosine phosphorylation of FAK and PXN induced by imatinib and nilotinib (Fig. 3A). Furthermore, treatment of U87MG cells with the FAK inhibitor PF-573,228 (PF-228) significantly reduced the imatinib and nilotinib stimulated increases in p130Cas and PXN tyrosine phosphorylation (Fig. 3B).

**Figure 4:**
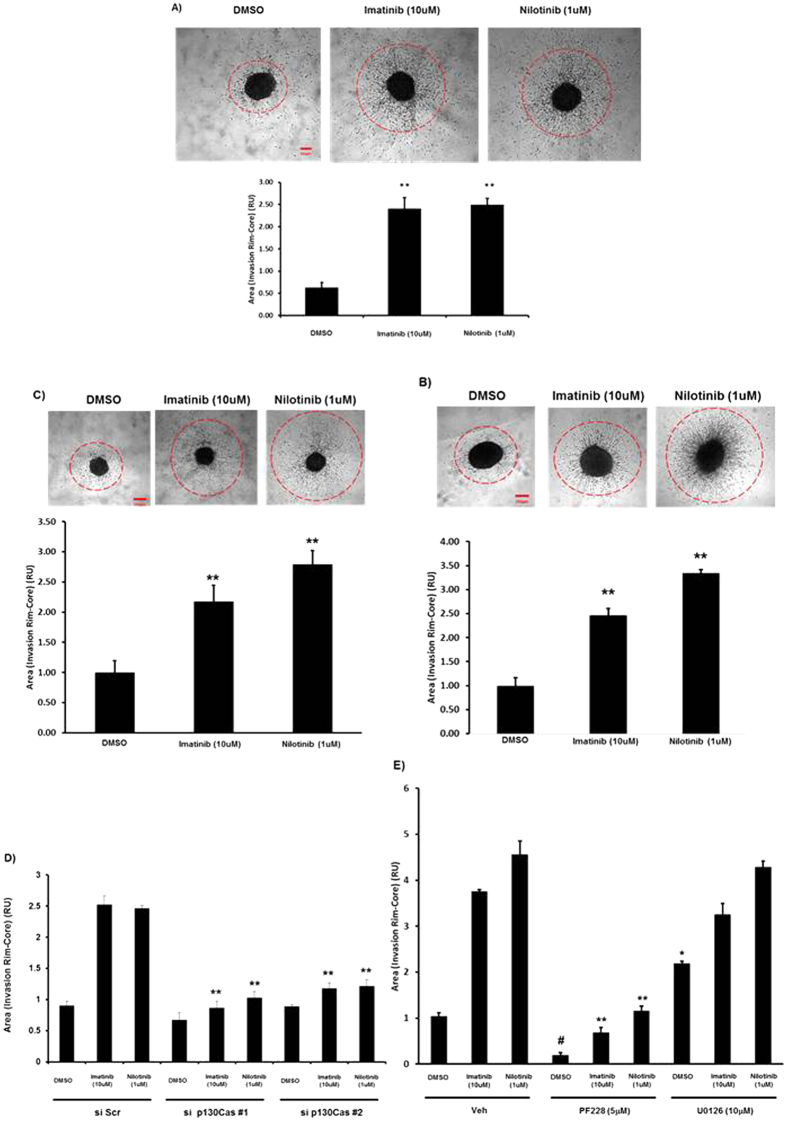
Imatinib and Nilotinib treatment of human GBM cells leads to increased 3D radial invasion of GBM cell spheroids (**A**) U87MG. (**B**) U251MG. (**C**) U118MG. (**A**–**C**) Equal amounts of cells were used to generate spheroids as described in Materials and Methods. 24 hours after spheroid production, spheroids were imbedded in a collagen gel and incubated in either SFM & DMSO vehicle control or 10 μM imatinib or 1 μM nilotinib for an additional 48 hours. **p < 0.01 compared to SFM & DMSO vehicle control. (**D**) Spheroids derived from U87MG cells treated with siRNA to p130Cas (si p130Cas #1 & #2) or control scrambled siRNA (siScr) were imbedded in collagen containing either SFM & DMSO vehicle control or 10 μM imatinib or 1 μM nilotinib and treated as above. **p < 0.01 compared to imatinib and nilotinib siScr respectively.(**E**) Spheroids derived from U87MG cells were imbedded in collagen containing either SFM & DMSO vehicle control, 5 μM PF573228 or 10 μM U0126 and treated as above. **p < 0.01 compared to imatinib and nilotinib DMSO vehicle control respectively. ^#^p>0.05 compared to SFM & DMSO vehicle control. Spheroids were fixed in 4% PFA and invasion was determined by measuring the area corresponding to the invasion rim minus the area of the core for at least 3 different spheroids per condition. Data from three independent experiments are presented as relative area units (RU) (means +/− s.e.m.).

**Figure 5:**
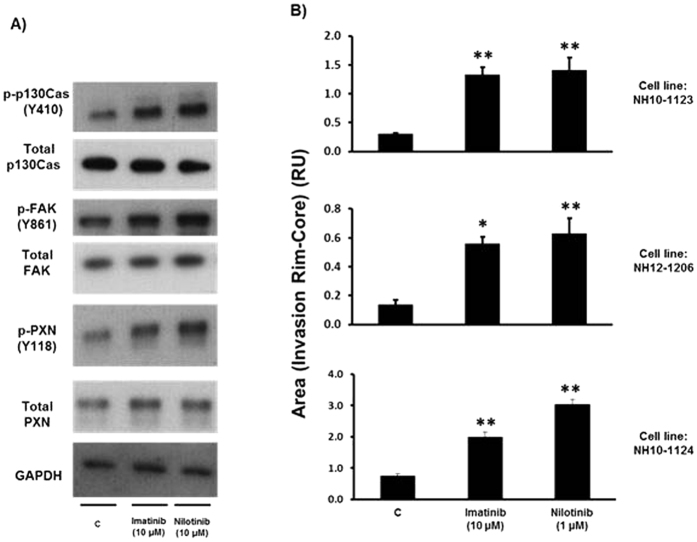
Imatinib and Nilotinib treatment of stem cells from human GBM biopsies leads to increased p130Cas, Focal Adhesion Kinase (FAK) and PXN tyrosine phosphorylation and increased 3D spheroid radial invasion. (**A**) Stem cells established from a human GBM biopsy (cell line: NH10-1124) (~80% confluent) were incubated in SFM for ~18 hr prior to treatment with either SFM & DMSO vehicle control (C), or imatinib or nilotinib for 20 minutes. Cell lysates were then prepared, blotted, and probed with the indicated antibodies. (**B**) Spheroids derived from the indicated stem cell lines established from human GBM biopsies were imbedded in a collagen gel and incubated in either SFM & DMSO vehicle control (C), or 10 μM imatinib or 1 μM nilotinib for an additional 48 hours. Spheroids were fixed in 4% PFA and invasion was determined by measuring the area corresponding to the invasion rim minus the area of the core for at least 3 different spheroids per condition. Data from three independent experiments are presented as relative area units (RU) (means +/− s.e.m.). *p < 0.05, **p < 0.01 compared to SFM & DMSO vehicle control (C).

Signalling via p130Cas and FAK pathways plays crucial roles in the regulation of cellular motility^13^,^20^,^24^. We therefore investigated the effects of imatinib and nilotinib treatment on glioma cell motility using both a three dimensional (3D) spheroid assay and two dimensional (2D) assays of chemotaxis and wound healing. We generated spheroids from U87MG, U251 and U118MG GBM cell lines and embedded them in collagen I plugs supplemented with either serum free medium (SFM) and DMSO (Veh), or SFM containing imatinib or nilotinib. Treatment with either imatinib or nilotinib alone for 48 hours resulted in a striking increase in radial invasion compared to the vehicle control spheroids (Fig. 4A–C). In transwell assays of chemotactic cell motility, we found surprisingly that imatinib acted as a chemo-attractant agent and was able to stimulate increased migration in U87MG cells (Fig. S6A). Furthermore, in wound healing assays in U251MG cells we found that either imatinib or nilotinib treatment resulted in increased rates of wound closure compared to control vehicle-treated cells (Fig. S6B). We next investigated the role of p130Cas, FAK and the MEK/ERK pathway in imatinib and nilotinib stimulated spheroid invasion. Spheroids from U87MG cells treated either with siRNA to p130Cas or with pharmacological inhibitors of FAK (PF-228) or MEK (U0126) were prepared and then treated with imatinib and nilotinib. Silencing of p130Cas, or treatment with PF-228 significantly reduced invasion induced by imatinib and nilotinib (Fig. 4D,E), whereas U0126 treatment had no significant effect on radial invasion in imatinib and nilotinib treated spheroids, although U0126 alone resulted in a small but significant increase in radial invasion compared with SFM vehicle control (Fig. 4E). The importance of cancer stem cells (CSCs) in tumour progression and their role in driving tumour relapse after treatment is emerging rapidly. GBM is one of the first tumours where (CSCs) were identified. Glioma stem cells (GSCs) are thought to be responsible for tumour maintenance and critically important in recurrence after resection^25^. We therefore examined the relevance of our findings in glioma cell lines to GSCs by investigating the effects of imatinib and nilotinib on GSCs isolated from human GBM biopsies. Imatinib and nilotinib treatment of a recently isolated GSC line significantly increased tyrosine phosphorylation of p130Cas, FAK and PXN (Fig. 5A). Furthermore, imatinib and nilotinib treatment of spheroids generated from the GSC line and two additional GSC lines significantly increased 3D radial invasion compared to the vehicle control (Fig. 5B).

## Discussion

Here we show that treatment of human glioma cell lines with the tyrosine kinase inhibitors imatinib and nilotinib produces a rapid and striking increase in tyrosine phosphorylation of p130Cas, FAK and PXN, key signalling molecules required for cell motility^20^,^24^. These effects were induced at concentrations similar to clinically relevant drug concentrations. The finding that imatinib and nilotinib treatment stimulates tyrosine phosphorylation as early as 10 minutes indicates that this is an immediate response though a direct drug effect on signalling components distal to p130Cas and FAK pathways. This is in contrast to the indirect result of longer-term effects on metabolism or gene expression^3^,^26^,^27^. Furthermore, localisation of tyrosine phosphorylated p130cas to the cell membrane in imatinib and nilotinib treated cells is in agreement with the previously reported roles for p130Cas in cell motility^20^.

A surprising result of this study is that the effects of imatinib and nilotinib on p130Cas, FAK and PXN tyrosine phosphorylation were independent of the major known tyrosine kinase targets for these drugs. Knockdown of ABL1, ABL2 or both ABL1 & ABL2 had no effect on imatinib and nilotinib stimulated tyrosine phosphorylation of p130Cas and PXN. We did however detect a significant reduction in imatinib and nilotinib stimulated tyrosine phosphorylation of FAK in cells treated with siRNA to ABL1, and both ABL1 & ABL2. This is most likely due to the reduction in total p130Cas expression observed in ABL1 and ABL1 & ABL2 treated cells. This is supported by our results showing that small changes in p130Cas knockdown efficiency between two different siRNAs exhibit differences in the reduced levels of imatinib and nilotinib stimulated tyrosine phosphorylation of FAK and Paxillin. Furthermore, knockdown of PDGFRβ, and DDR1 had no direct effect on imatinib and nilotinib stimulated tyrosine phosphorylation of p130Cas, FAK and PXN. Whilst the lack of expression of c-Kit in U87MG cells indicates it is not responsible for these effects. It is important to note that these experiments also preclude the possibility of kinase independent roles for these targets. SRC is the main kinase responsible for tyrosine phosphorylation of these proteins^20^. We found that pharmacological inhibition of SRC leads to a complete abrogation of both basal and imatinib/nilotinib induced tyrosine phosphorylation of p130Cas, FAK and PXN. However, imatinib and nilotinib treatment did not result in any changes in the levels of tyrosine 416 phosphorylation in SRC, making it difficult to interpret these data in the context of a direct role for SRC.

Another potential mechanism underlying the effects of imatinib and nilotinib on motility-associated tyrosine phosphorylation is increased signalling through the RAF/MEK/ERK pathway, which has recently been reported to occur in several human tumour cell lines in response to these drugs^3^. Whilst we observed increased ERK phosphorylation in response to imatinib treatment, nilotinib treatment did not lead to increased ERK phosphorylation, possibly pointing to engagement of different signalling pathways in glioma cells. Furthermore, inhibition of MEK/ERK signalling using U0126 had no effect on imatinib- and nilotinib-stimulated tyrosine phosphorylation. In addition, we observed no effect of silencing of B-RAF or C-RAF, either alone or together, on increased tyrosine phosphorylation in response to imatinib and nilotinib.

Many studies have shown a role for integrin receptors and integrin signalling in the regulation of p130Cas, FAK and PXN tyrosine phosphorylation either through “inside-out” or “outside-in” signalling^20^,^24^. We however, found no effect on increased tyrosine phosphorylation in cells which were depleted of integrin β1 or Integrin β3 by gene silencing. This result is in agreement with our recent work showing that p130Cas tyrosine phosphorylation and 3D invasion were independent of integrin β1 in U87MG cells^28^.

Both p130Cas and FAK have been shown to signal through multi-protein complexes, required for cell motility^13^,^20^, yet the individual contributions of these molecules in such complexes is not completely understood. Our previous studies show that platelet derived growth factor (PDGF-BB) or hepatocyte growth factor (HGF) stimulated tyrosine phosphorylation of p130Cas in U87MG cells was independent of FAK kinase activity^12^. However, we show here that both p130Cas expression and FAK kinase activity are required for the increased tyrosine phosphorylation of p130Cas, FAK, and PXN, and glioma cell spheroid invasion induced by imatinib and nilotinib. This supports the conclusion that imatinib and nilotinib impact upon p130Cas and FAK complexes via a signalling pathway distinct from that mediating PDGFRβ and c-Met signalling and motility in U87MG cells^12^.

An important finding of this study is that, consistent with increased tyrosine phosphorylation of p130Cas, FAK, and PXN induced by imatinib and nilotinib treatment, these drugs strikingly increased motility in 2D and 3D models using multiple human glioma cell lines. The conclusion that the pro-invasive activity of imatinib and nilotinib is mediated through p130Cas and FAK signalling is supported by the finding that the response to these drugs was strongly reduced by silencing of p130Cas and inhibition of FAK, but not by the inhibition of MEK.

Imatinib and nilotinib are successfully used in the clinic as front line therapies for Ph+ CML. In this context, Ph+ CML is the result of a single driver mutation and does not possess the genetic heterogeneity that is observed in GBM^29^. CML is a cancer of the white blood cells, resulting in increased and unregulated growth of predominantly myeloid cells in the bone marrow and the accumulation of these cells in the blood^30^. These cells are non-adherent and do not rely on adherent modes of cell motility for tumour progression. Imatinib-resistant Ph+ CML patients are either initially resistant (primary) or develop resistance over the course of treatment (acquired)^31^. Patients with advanced stage disease frequently have both^32^. The process involved in both primary and acquired imatinib-resistance can be divided into either BCR-ABL-dependent or BCR-ABL -independent mechanisms. BCR-ABL-dependent mechanisms include point mutations within the kinase domain resulting in reduced drug potency, while BCR-ABL -independent mechanisms are more varied and poorly understood. However, there are reports of imatinib-resistant Ph+ CML tumour cells undergoing a mesenchymal-like conversion associated with increased levels of FAK tyrosine phosphorylation and increased adherence and invasiveness^26^. Furthermore, imatinib is used as an adjuvant therapy in patients with Gastrointestinal stromal tumour (GIST) and there are reports of imatinib treated GIST cells showing increased FAK tyrosine phosphorylation after long term treatment (6–24 hours)^27^,^33^. On the basis of our results, we also propose that pharmacological modulation of FAK activity could provide an approach to boost imatinib and nilotinib efficacy and limit resistance to these drugs in CML & GIST and other cancers in which imatinib is used, a possibility that warrants further investigation. In this context, it is of interest that pharmacological inhibition of FAK using TAE226 can synergise with nilotinib in reducing Ph+ CML growth^34^,^35^. Furthermore, a FAK-selective inhibitor (TAG372) induced apoptosis of imatinib-resistant GIST-T1 cells and decreased the imatinib IC50^33^.

The results presented here suggest a potential adverse effect of the use of imatinib and nilotinib treatment in GBM tumours, which is dependent on augmented adherent tumour cell motility. The relevance of our study for human GBM is further underscored by the finding that imatinib and nilotinib treatment of stem cell lines derived from human GBM biopsies also increased p130Cas, FAK and PXN tyrosine phosphorylation and radial invasion of spheroids generated from these cell lines. Imatinib and nilotinib treatment resulting in enhanced invasion through increased p130Cas and FAK signalling could lead to selection of tumour cells with increased motility independent of c-ABL and PDGFRβ signalling pathways. Recently, imatinib was tested in a multi-centre phase III clinical trial for patients with recurrent GBM. The results indicated that there were no clinically meaningful differences between mono therapies (Hydroxy Urea or Temozolimide) or combination therapies with imatinib^36^ and the primary study end point was not met. GBM is an extremely aggressive cancer due in part to its highly invasive nature. Our findings indicate important and unforeseen adverse effects of imatinib and nilotinib treatment on tumour cell motility, which could be a significant contributor to the lack of clinical efficacy observed in these trials. These results point to the necessity for screening imatinib, nilotinib and other members of this class of TKI for their effects on multiple modes of cell motility in specific cancers and other diseases where therapeutic efficacy is being investigated.

## Additional Information

**How to cite this article**: Frolov, A. *et al*. Imatinib and Nilotinib increase glioblastoma cell invasion via Abl-independent stimulation of p130Cas and FAK signalling. *Sci. Rep.* **6**, 27378; doi: 10.1038/srep27378 (2016).

## Supplementary Material

Supplementary Information
