# Supplementary material for: Imatinib and Nilotinib increase glioblastoma cell invasion via Abl-independent stimulation of p130Cas and FAK signalling
Source: Sci Rep. 2016 Jun 13;6:27378. doi: 10.1038/srep27378 (PMC4904410; doi:10.1038/srep27378)
Supplement: Supplementary Information [file srep27378-s1.pdf]

Imatinib and Nilotinib increase glioblastoma cell invasion via Abl-independent stimulation of p130Cas and FAK signalling.

Antonina Frolov, Ian M. Evans, Ningning Li, Kastytis Sidlauskas, Ketevan Paliashvili, Nicola Lockwood, Angela Barrett, Sebastian Brandner, Ian C. Zachary and Paul Frankel

Supplementary Figure Legends

Supplementary Figure S1: Imatinib and nilotinib treatment of U251MG cells leads to increased p130Cas, Focal Adhesion Kinase (FAK) and Paxillin (PXN) tyrosine phosphorylation. Cells (~80% confluent) were incubated in SFM for ~18hr prior to treatment with vehicle control (C), or 10 μM Imatinib or 10 μM Nilotinib for 20 minutes.
Cell lysates were then prepared, blotted, and probed with the indicated antibodies.

Supplementary Figure S2: DDR1 & c-Kit are not required for Imatinib and nilotinib stimulated increases in p130Cas, FAK and PXN tyrosine phosphorylation.
(**A**), U87MG cells were transfected with siRNA targeting DDR1 (siDDR1), cells were transfected at a concentration of 25 nM, or with 25 nM of a control scrambled siRNA (siScr). 48hr post transfection, cells were incubated in serum-free medium (SFM) for ~18hr prior to treatment with SFM & DMSO vehicle control (C), or 10 μM Imatinib or 10 μM Nilotinib for 20 minutes. Cell lysates were then prepared, blotted, and probed with the indicated antibodies. (**B**), U87MG cells, Human umbilical vein endothelial cells (HUVECs) and Human smooth muscle cells (HCASMCs) were blotted for c-Kit and GAPDH (as a loading control). Only the smooth muscle cells expressed detectable levels of c-Kit.

Supplementary Figure S3: Increased p130Cas, FAK and Paxillin tyrosine phosphorylation is dependent on SRC kinase activity. U87MG cells (~80% confluent) were incubated in SFM for ~18hr prior to pre-incubation for 20 min with 10 μM PP2 or the vehicle (0.05% DMSO) (C) prior to treatment with SFM & DMSO vehicle control (C), or 10 μM imatinib or 10 μM nilotinib for 20 minutes. Cell lysates were then prepared, blotted, and probed with the indicated antibodies.

Supplementary Figure S4: Increased p130Cas, FAK and PXN tyrosine phosphorylation is independent of RAS / RAF/ MEK / MAPK and Integrin signalling pathways
(**A**) U87MG cells (~80% confluent) were incubated in SFM for ~18hr prior to pre-incubation for 30 min with 10 μM U0126 or the vehicle (0.05% DMSO) (C) prior to treatment with SFM & DMSO vehicle control (C), or 10 μM imatinib or 10 μM nilotinib for 20 minutes. (**B**) U87MG cells were transfected with siRNA targeting BRAF (siBRAF), CRAF (siCRAF) or together at a concentration of 25 nM, or with 25 nM of a control scrambled siRNA (siScr). (**C & D**) U87MG cells were transfected with siRNA targeting Integrin β1 (siITGB1) or Integrin β3 (siITGB3) at a concentration of 25 nM, or with 25 nM of a control scrambled siRNA (siScr).

48hr post transfection, cells were incubated in serum-free medium (SFM) for ~18hr prior to treatment with SFM & DMSO vehicle control (C), or 10 µM imatinib or 10 µM nilotinib for 20 minutes. Cell lysates were then prepared, blotted, and probed with the indicated antibodies.

Supplementary Figure S5: Co-immunoprecipitation of p130Cas, FAK, and Paxillin in U87MG cells. DMSO, Imatinib or Nilotinib treated cells were immunoprecipitated with p130Cas antibody. Immunoprecipitates were blotted, and probed with the indicated antibodies.

Supplementary Figure S6: Imatinib and nilotinib treatment leads to increased 2D cell motility in GBM cells. (**A**) U87MG cells were used in a Transwell migration assay as detailed in Experimental. Values ($n \geq 3$) are means ± s.e.m, expressed as the number of cells migrating per field; *$p<0.01$ compared to vehicle treated control (C). (**B**) U251 cells were grown to confluence in a 96-well plate and a uniform scratch was made along the centre of each well. Cells were washed with serum free medium and incubated with either 10 µM Imatinib, 1 µM Nilotinib or DMSO control in serum free medium. Rate of wound closure was monitored in an Incucyte Zoom with images captured every 2 hours for a total of 30 hours.

Supplementary Figure S7: Imatinib and nilotinib treatment does not effect U87MG cell proliferation. Proliferation was measured using U87MG cells (stably expressing H2B-GFP) as detailed in Materials and Methods. Values are means from 5 replicate wells per experiment and 5 independent experiments ($n = 5$) ± s.e.m, expressed as the relative fluorescence intensity per well.

Supplementary Figure S1

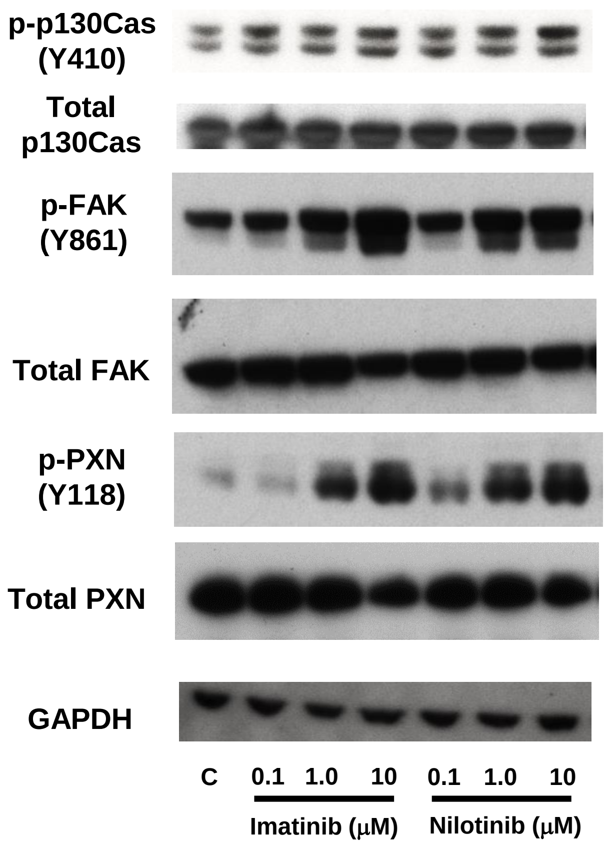

Frolov, et al.

Supplementary Figure S2

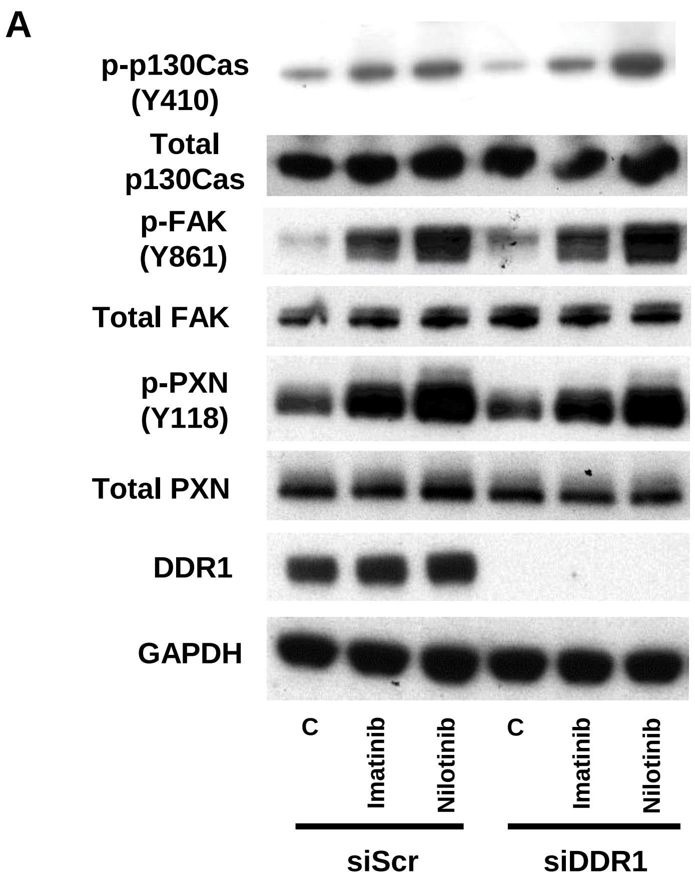

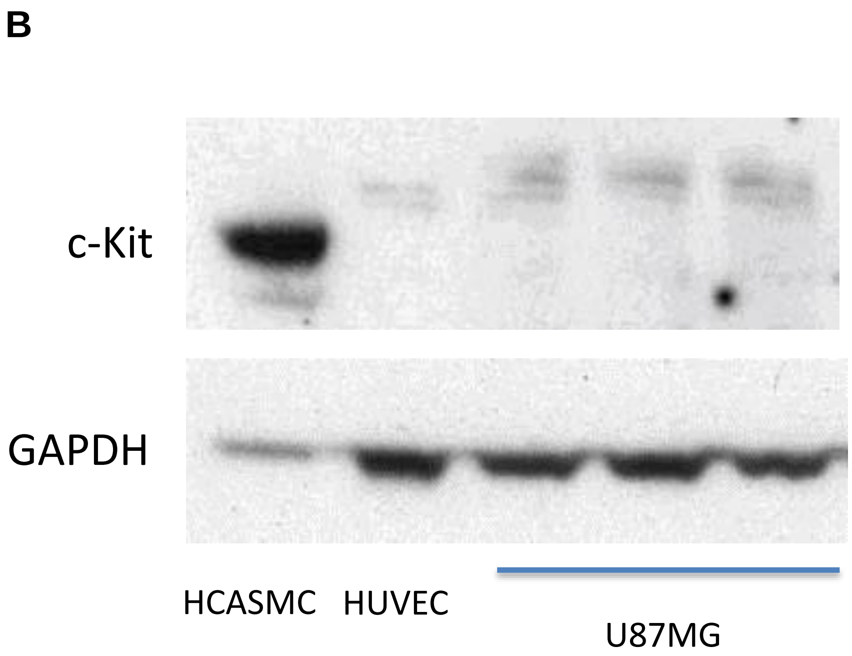

Frolov, et al.

Supplementary Figure S3

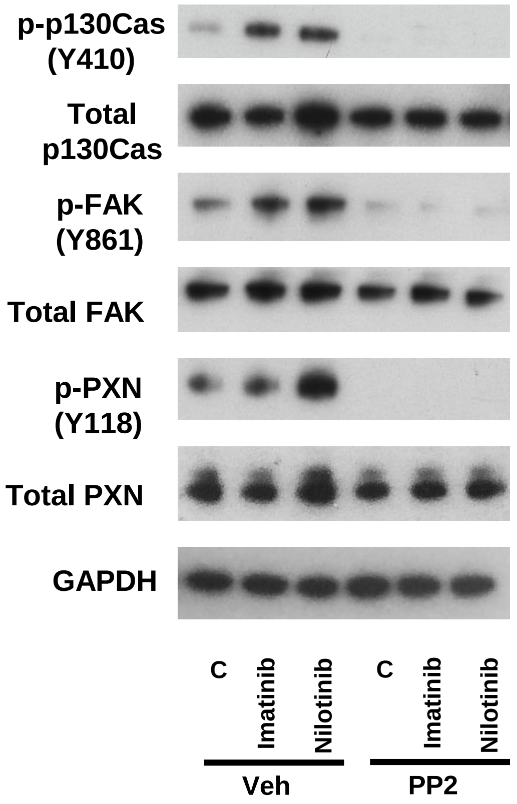

Frolov, et al.

Supplementary Figure S4

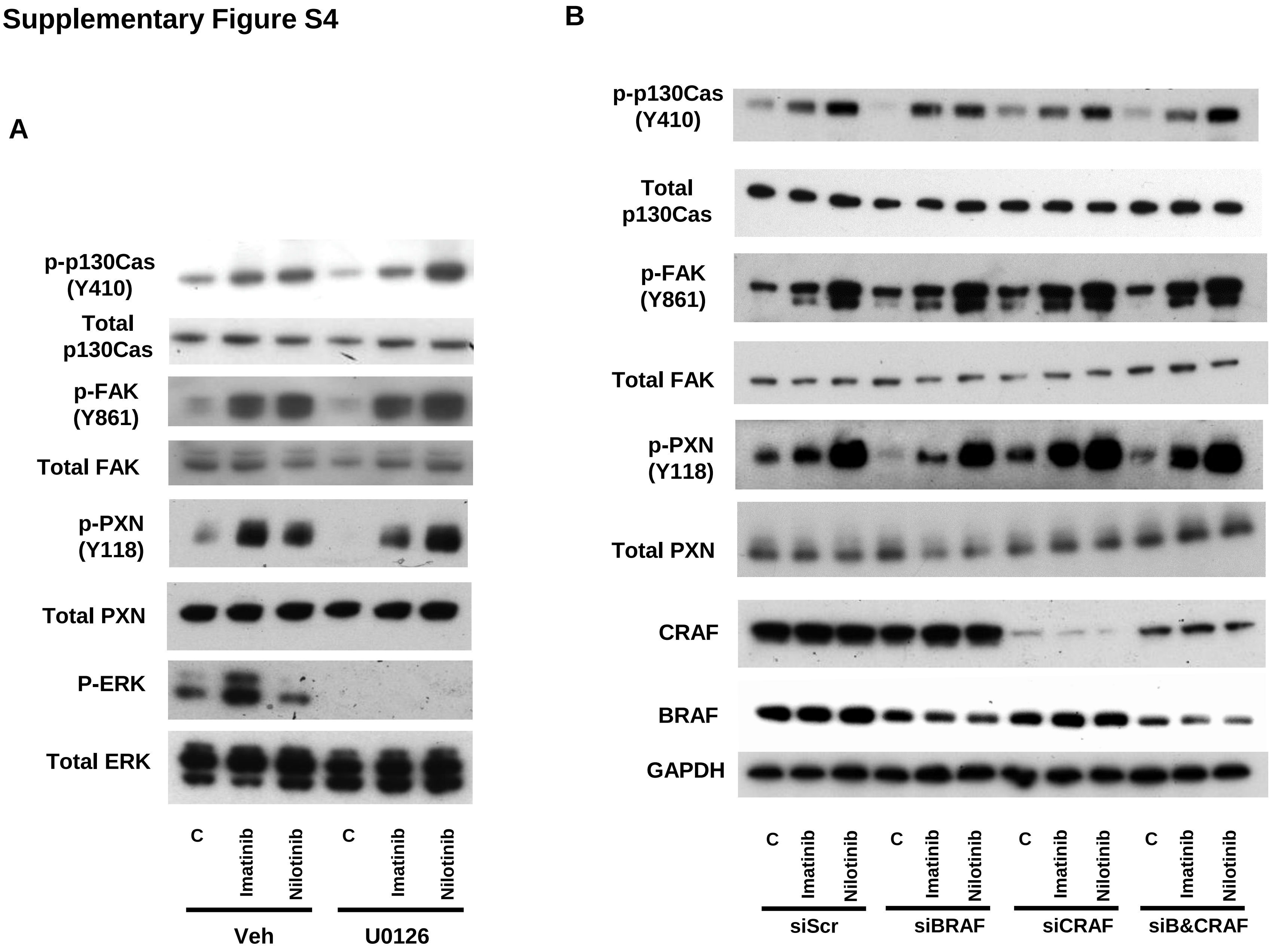

Frolov, et al.

Supplementary Figure S4

C

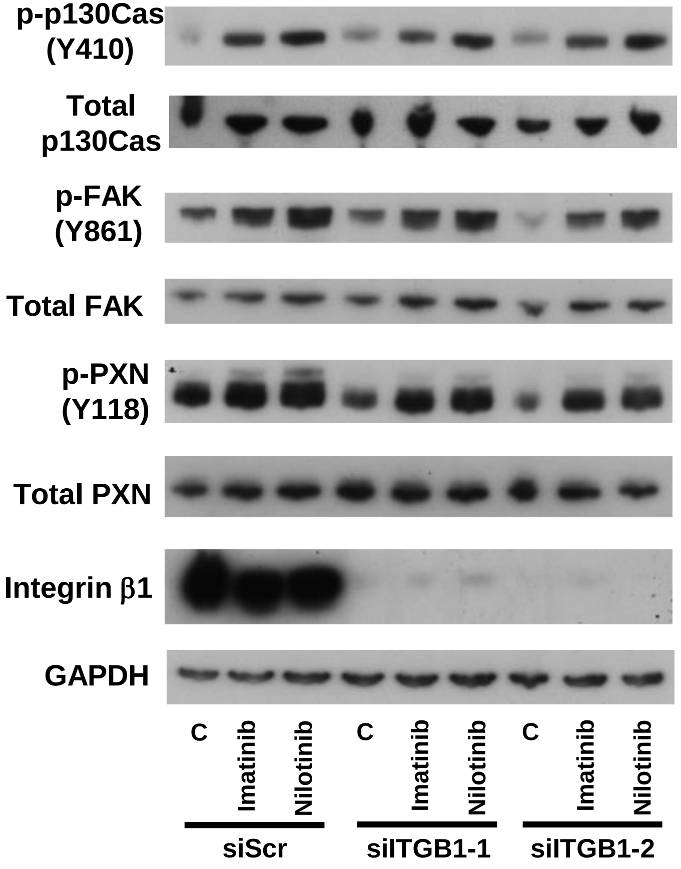

D

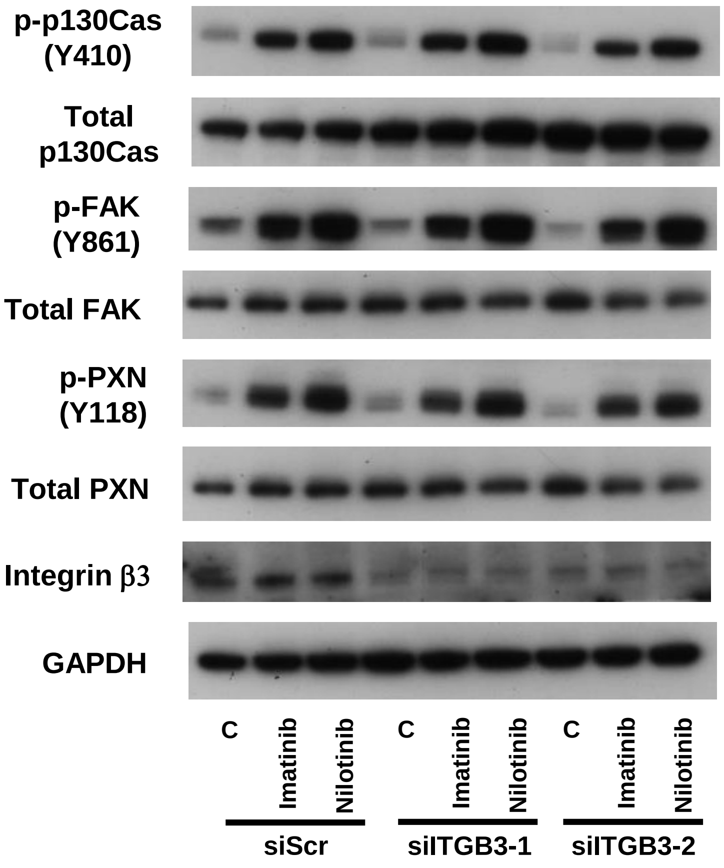

Frolov, et al.

Supplementary Figure S5

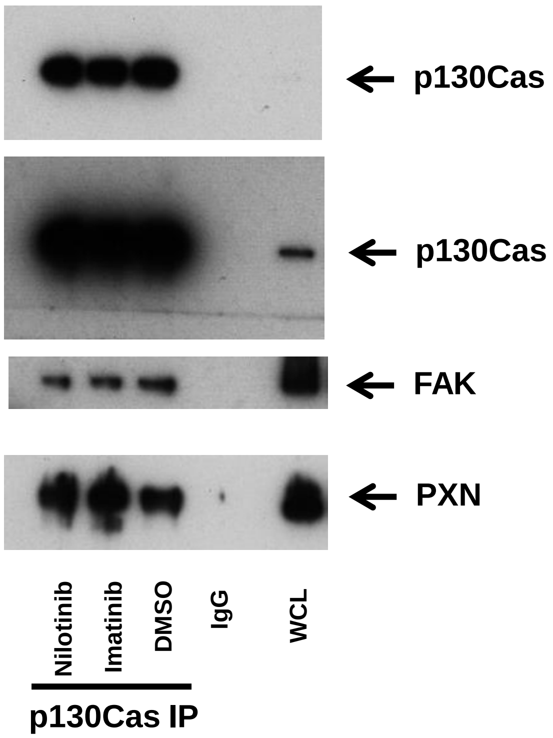

Frolov, et al.

Supplementary Figure S6A

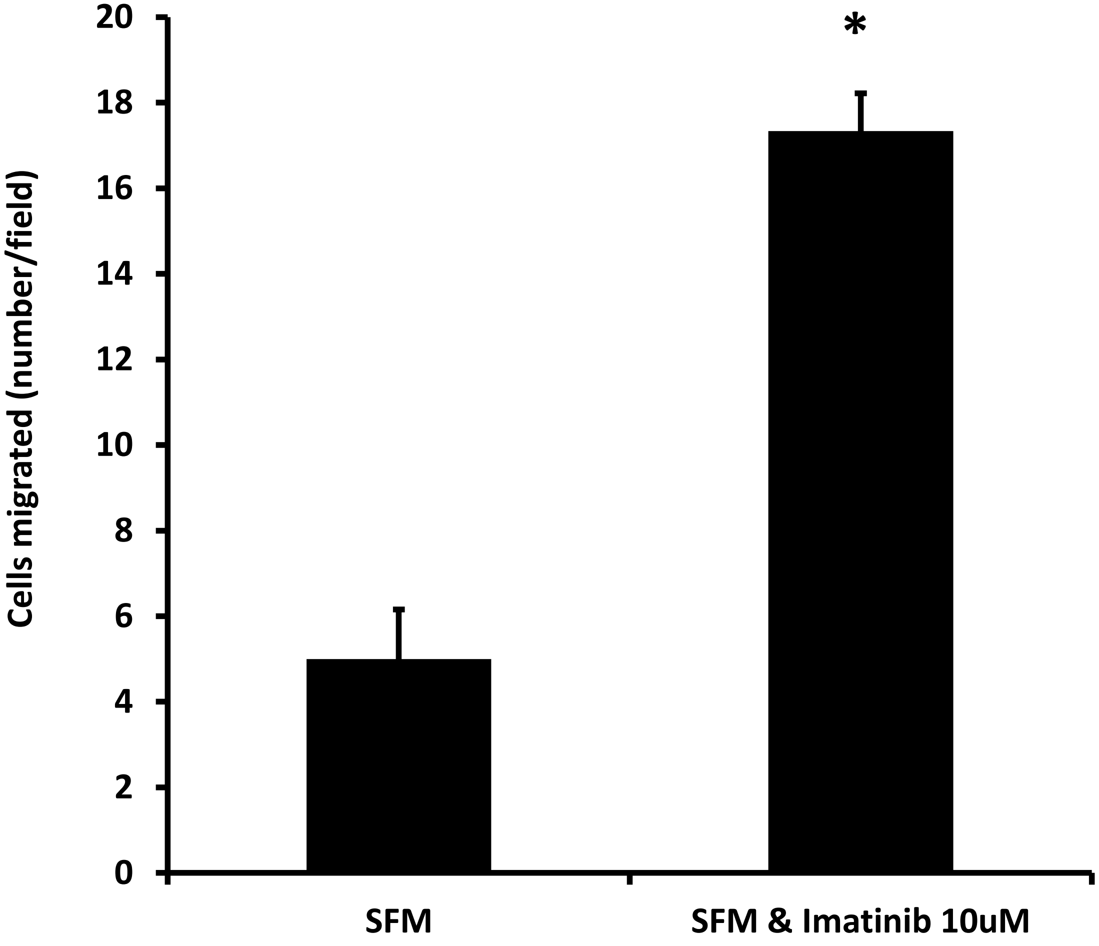

Frolov, et al.

Supplementary Figure S6B

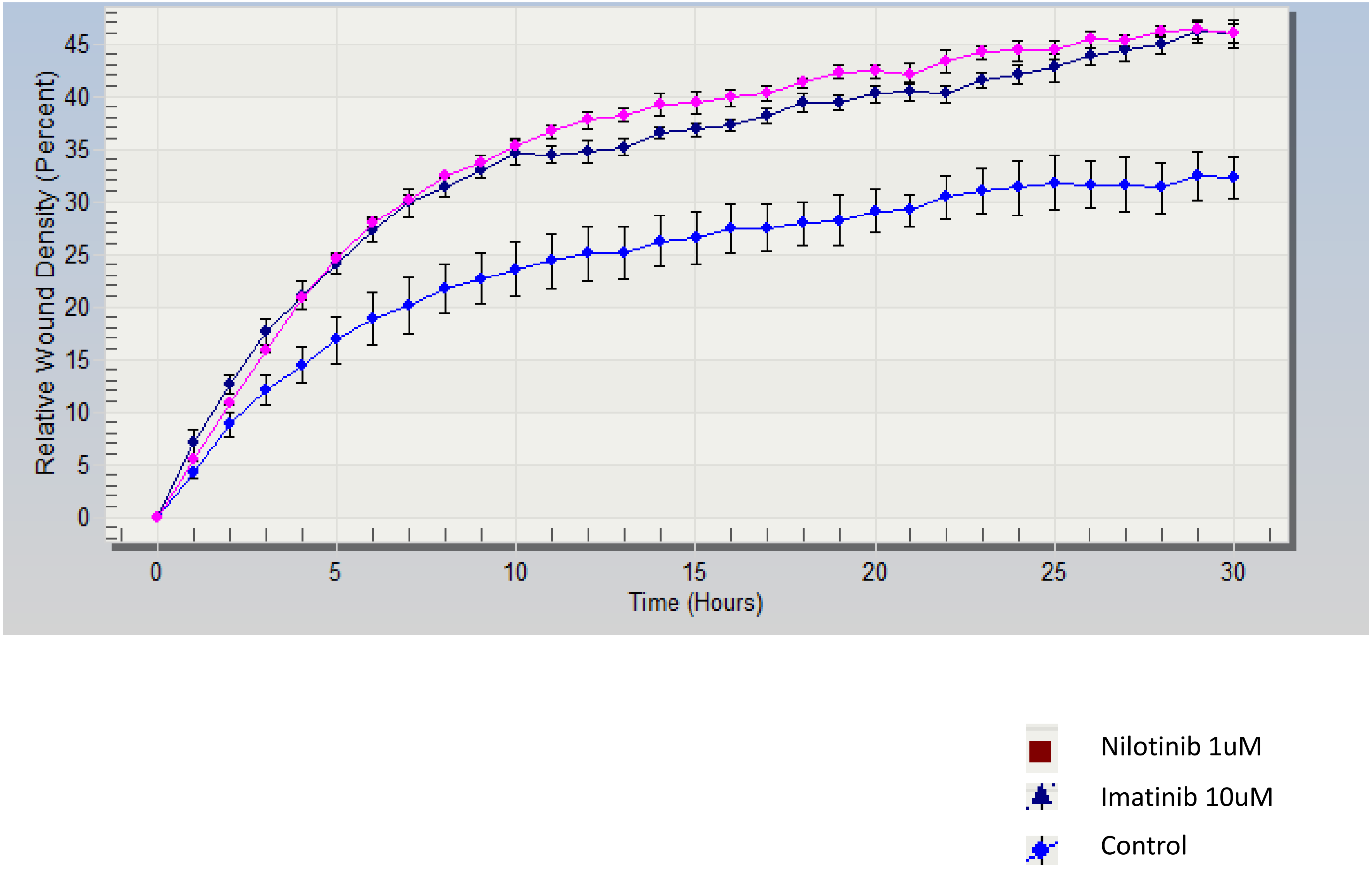

Frolov, et al.

Supplementary Figure S7

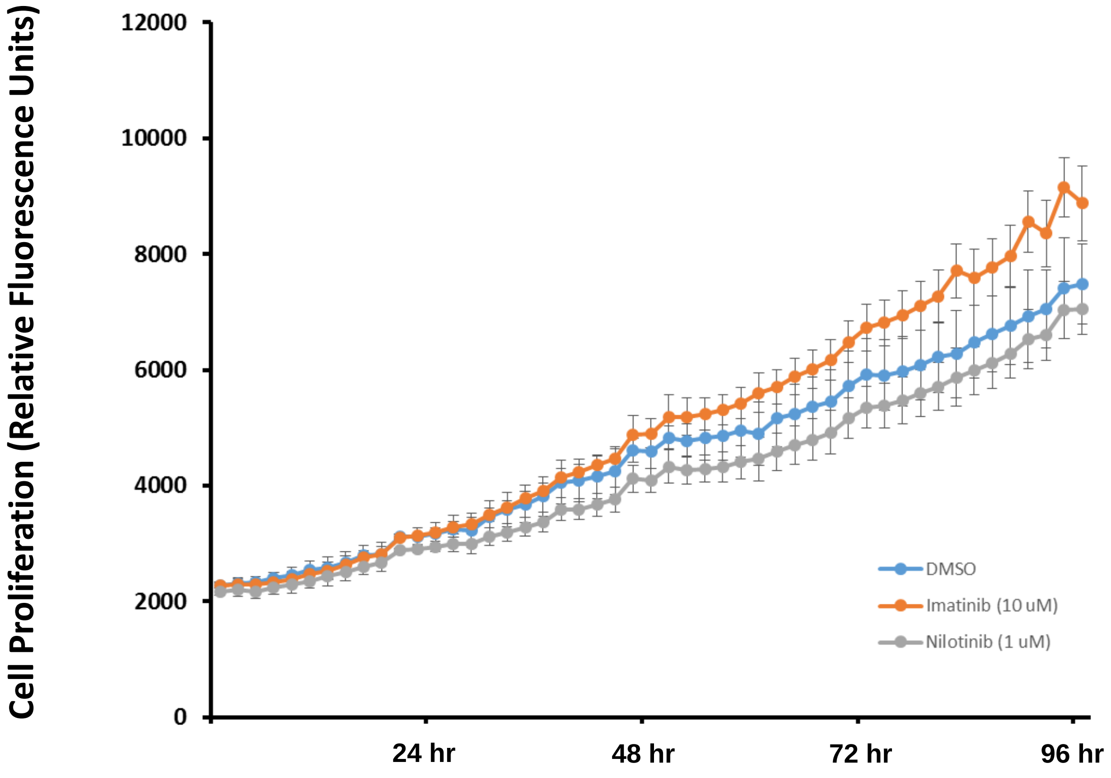

Frolov, et al.
